# Dynamic 5-HT_2C_ Receptor Editing in a Mouse Model of Obesity

**DOI:** 10.1371/journal.pone.0032266

**Published:** 2012-03-20

**Authors:** Harriët Schellekens, Gerard Clarke, Ian B. Jeffery, Timothy G. Dinan, John F. Cryan

**Affiliations:** 1 Food for Health Ireland, University College Cork, Cork, Ireland; 2 Laboratory of Neurogastroenterology, Alimentary Pharmabiotic Centre, University College Cork, Cork, Ireland; 3 Department of Psychiatry, University College Cork, Cork, Ireland; 4 Department of Anatomy and Neuroscience, University College Cork, Cork, Ireland; 5 School of Pharmacy, University College Cork, Cork, Ireland; 6 Department of Microbiology, University College Cork, Cork, Ireland; University of Minnesota, United States of America

## Abstract

The central serotonergic signalling system has been shown to play an important role in appetite control and the regulation of food intake. Serotonin exerts its anorectic effects mainly through the 5-HT_1B_, 5-HT_2C_ and 5-HT_6_ receptors and these are therefore receiving increasing attention as principal pharmacotherapeutic targets for the treatment of obesity. The 5-HT_2C_ receptor has the distinctive ability to be modified by posttranscriptional RNA editing on 5 nucleotide positions (A, B, C, D, E), having an overall decreased receptor function. Recently, it has been shown that feeding behaviour and fat mass are altered when the 5-HT_2C_ receptor RNA is fully edited, suggesting a potential role for 5-HT_2C_ editing in obesity. The present studies investigate the expression of serotonin receptors involved in central regulation of food intake, appetite and energy expenditure, with particular focus on the level of 5-HT_2C_ receptor editing. Using a leptin-deficient mouse model of obesity (*ob/ob*), we show increased hypothalamic 5-HT_1A_ receptor expression as well as increased hippocampal 5-HT_1A_, 5-HT_1B_, and 5-HT_6_ receptor mRNA expression in obese mice compared to lean control mice. An increase in full-length 5-HT_2C_ expression, depending on time of day, as well as differences in 5-HT_2C_ receptor editing were found, independent of changes in total 5-HT_2C_ receptor mRNA expression. This suggests that a dynamic regulation exists of the appetite-suppressing effects of the 5-HT_2C_ receptor in both the hypothalamus and the hippocampus in the *ob/ob* mice model of obesity. The differential 5-HT_1A_, 5-HT_1B_ and 5-HT_6_ receptor expression and altered 5-HT_2C_ receptor editing profile reported here is poised to have important consequences for the development of novel anti-obesity therapies.

## Introduction

Obesity is rapidly increasing in prevalence in developed countries [1], [2]. Thus, there is increasing medical and societal needs for novel treatments, which induce appetite suppression and weight loss. Satiety and appetite control pathways have been studied extensively both in animals and humans but the exact underlying molecular mechanisms remain unclear [3], [4], [5], [6]. It is well established that increased serotonin (5-hydroxytryptamine, 5-HT) neurotransmission in the brain regulates food intake [7], [8], [9]. In particular, 5-HT_1B_, 5-HT_2C_ and 5-HT_6_ receptors have received attention as promising anti-obesity therapeutic targets [10], [11], [12], [13], [14], [15]. Centrally acting serotonergic agents, including sibutramine, m-chlorophenylpiperazine (mCPP) and fenfluramine, act as potent appetite suppressants [16], [17], [18]. However, these compounds are pharmacologically promiscuous, showing activity across multiple 5-HT and non-5-HT pathways and receptors, and accordingly exert many unwanted side effects. A better understanding of the mechanisms by which serotonergic receptors regulate appetite and energy homeostasis may lead to the development of novel effective anti-obesity drugs.

**Figure 1:**
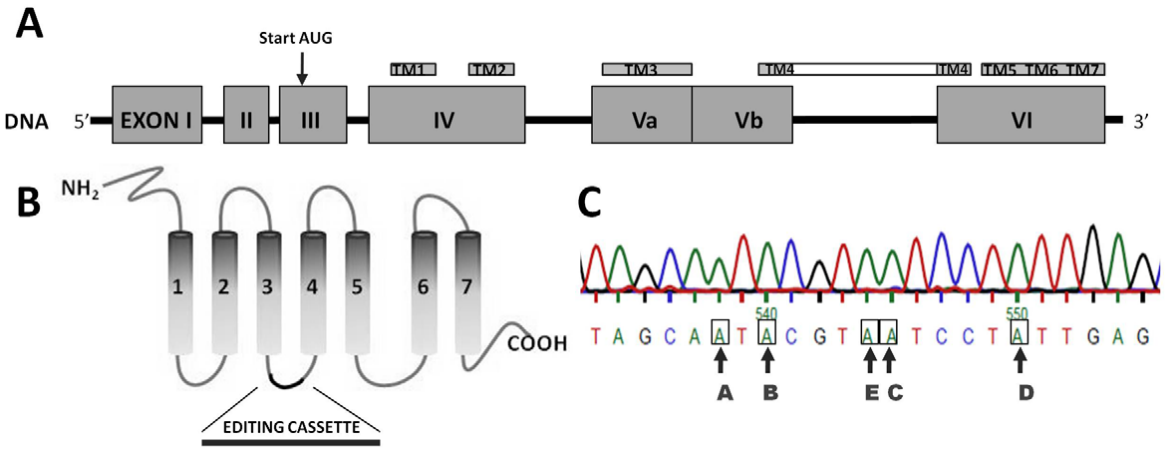
Serotonin 2C receptor gene structure. A) The human full-length 5-HT_2C_ gene, located on the X chromosome and processed from mRNA encoded from exon 3 to exon 6 after splicing out intronic sequence is depicted (not including 3′- or 5′- untranslated regions and not according to scale). B) The 5-HT_2C_ gene is translated into a seven-transmembrane G-protein coupled receptor. The editing cassette is located in the second intracellular loop. C) The nucleotide sequence of the 5-HT_2C_ editing cassette is depicted including the five nucleotide positions prone to adenosine to inosine editing.

Within the serotonergic system, the 5-HT_2C_ receptor requires special attention due to its distinctive ability to be modified by post-transcriptional RNA editing [19]. The 5-HT_2C_ receptor pre-RNA can be enzymatically edited on 5 specific nucleotide positions (A, B, C, D, E) converting an adenosine to inosine residues, causing amino acid sequence changes. Selective editing can generate up to 32 different mRNA isoforms translating into 24 predicted protein sequences, all with unique signalling features (Figure 1). Even though not all 5-HT_2C_ isoforms have been tested to date, it is accepted that increased RNA editing reduces receptor constitutive activity and decreases agonist potency and G-protein coupling, resulting in an overall decreased receptor function [19], [20], [21], [22], [23], [24], [25]. In addition, distribution of edited 5-HT_2C_ isoforms has been shown to be different across brain regions [26]. Therefore, differential editing of the 5-HT_2C_ receptors in the CNS may have important consequences for the functional properties of the receptor *in vivo*. Recently, it has been shown that feeding behaviour and fat mass are altered when studying mice engineered to express a fully edited 5-HT_2C_ receptor isoform in the brain, suggesting a potential role for 5-HT_2C_ receptor editing in obesity [24], [27], [28]. In addition, 5-HT_2C_ RNA editing status has been implicated in psychiatric and stress-related disorders and has been shown to be a dynamic process, demonstrating changes in response to either stress or pharmacotherapeutic drug across *in vitro* and *in vivo* studies [26], [29], [30], [31], [32], [33], [34], [35]. The 5-HT_2C_ receptor RNA editing profile within obesity phenotypes and its impact on feeding has so far, to our knowledge not been investigated. The *ob/ob* mouse, a leptin protein deficient strain, is one of the most widely used mouse model of obesity and is characterised by several metabolic and neuroendocrine abnormalities, including a prominent hyperphagia leading to obesity [36], [37], [38], [39]. This study aims to analyse central mRNA expression levels of 5-HT receptors related to feeding (5-HT_1A_, 5-HT_1B_, 5-HT_6_, 5-HT_2C_) within this mouse model of obesity (*ob/ob*) and in particular to analyse if there is an altered 5-HT_2C_ receptor editing profile within the obesity phenotype, by analysing the expression of partially as well as fully edited 5-HT_2C_ receptor isoforms.

## Materials and Methods

### Animals

Animals, male *ob/ob* mice (n = 8–10 per cohort) and lean littermate controls (n = 8–10 per cohort), generated on a C57BL/6 background, were purchased from Harlan, UK. The sample size is based on a power calculation aimed at detecting differences at the 0.05 level. Mice were received at the facility when they were 5 to 6 weeks old. Groups of four mice were housed in standard holding cages in a light-controlled (12-hour light/dark cycle; lights on at 7.45 am), temperature-controlled (21°C±1) and humidity-controlled (55±10%) environment. Water was available *ad libitum* throughout the study and 10 g pre-weighed standard lab chow (2018S Teklad Global 18% Protein Rodent Diet) was given per mouse each day. Mice were weighed each day between 9am and 10am and the amount of daily food intake was calculated. Animals were sacrificed at ages between 8 and 9 weeks using cervical dislocation. Brain tissue was dissected at 4°C, processed in RNA Later (Ambion, Warrington, UK) and stored at −80°C until the analysis. The hypothalamus and hippocampus were the two regions where most of the analysis is carried out. The brains were removed from the skull and placed with ventral side up on an ice-cooled Petri dish. For dissection, the coordinates of the brain regions were selected according to the “The Mouse Brain in Stereotaxic Coordinates, 3rd Edition” [40]. Using a curved forceps, the hypothalamus was pinched out from the ventral surface of the brain by pushing the curved part of the forceps down around the hypothalamus starting directly behind the optic chiasm. With the dorsal side up, a sagittal cut was made down the midline of the brain, leaving the cerebellum and brainstem intact. The hippocampai were separated from the white matter beneath the neocortex with a curved forceps and pinched out from each side of the brain. All daytime samples were harvested in the morning, directly following the dark phase. In addition, hypothalamus brain tissue was also harvested from a different cohort of animals in the evening, before onset of the dark phase, designated as nighttime samples. All experiments were conducted in full accordance with the European Community Council Directive 86/609/EEC, the Recommendation 2007/526/65/EC and approved by the Animal Experimentation Ethics Committee of University College Cork (Animal ethical permit number #2010/028). All efforts were made to minimise animal suffering and to reduce the number of animals used. All experiments in this manuscript are performed on the same cohort of animals, with the exception of the neurotransmitter concentration determination.

### Neurotransmitter concentrations

Neurotransmitter concentrations were determined in *ob/ob* mice and control littermates, using a modification of a previously described procedure [41]. Briefly, brain tissue was sonicated in 500 µl of chilled mobile phase spiked with 4 ng/40 ul of N-Methyl 5-HT (Sigma Chemical Co., UK) as internal standard. The mobile phase contained 0.1 M citric acid, 5.6 mM octane-1-sulphonic acid (Sigma), 0.1 M sodium dihydrogen phosphate, 0.01 mM EDTA (Alkem/Reagecon, Cork) and 9% (v/v) methanol (Alkem/Reagecon), and was adjusted to pH 2.8 using 4N sodium hydroxide (Alkem/Reagecon). Homogenates were then centrifuged for 15 minutes at 14,000 rpm at 4°C and 40 µl of the supernatant injected onto the HPLC system which consisted of a SCL 10-Avp system controller, LECD 6A electrochemical detector (Shimadzu), a LC-10AS pump, a CTO-10A oven, a SIL-10A autoinjector (with sample cooler maintained at 40C) and an online Gastorr Degasser (ISS, UK). A reverse-phase column (Kinetex 2.6 u C18 100×4.6 mm, Phenomenex) maintained at 30°C was employed in the separation (Flow rate 0.9 ml/min). The glassy carbon working electrode combined with an Ag/AgCL reference electrode (Shimdazu) was operated a +0.8V and the chromatograms generated were analysed using Class-VP 5 software (Shimadzu). The neurotransmitters were identified by their characteristic retention times as determined by standard injections, which were run at regular intervals during the sample analysis. The ratios of peak heights of analyte versus internal standard were measured and compared with standard injection. Results were expressed as ng of neurotransmitter per g fresh weight of tissue.

### Sample preparation

Total RNA was isolated using the Absolutely RNA® Miniprep kit (Stratagene, La Jolla, USA) according to manufacturer's instructions. Briefly, brain tissues were homogenized using a Polytron PT2100 in RNA lysis buffer and nucleic acids were extracted using a buffer and spin column protocol. The nucleic acids were subsequently washed and separated using an elution column. DNase treatment was carried out using the Ambion Turbo DNase kit (Ambion, Warrington, UK) according to manufacturer's instructions. RNA was quantified using NanoDrop™ spectrophotometer (Mason Technology, Cork, Ireland) according to the manufacturer's instructions. RNA quality and RNA integrity number (RIN) were determined using the Agilent™ Bioanalyzer (Agilent, Stockport, UK). RNA samples that satisfied criteria (RIN value >7) were reverse transcribed to cDNA using the High Capacity cDNA kit (Applied Biosystem, Warrington, UK) according to manufacturer's protocol. Briefly, Multiscribe Reverse Transcriptase (50 U/µL) was added as part of the RT master mix, incubated at 25°C for 10 minutes, at 37°C for 2 hours, at 85°C for 5 minutes and stored at 4°C.

### Real-time quantitative RT-PCR

Quantitative PCR (Q-PCR) was carried out using 6 carboxy fluorescein (FAM™) dye-labeled TaqMan® MGB probes supplied by Applied Biosystems™ to mouse specific 5-HT_1A_, 5-HT_1B_, total 5-HT_2C_, full-length 5-HT_2C_, 5-HT_6_, ADAR1 and ADAR2 while using β-Actin as an endogenous control (Mm00434106_s; Mm00439377_s1; Mm00434127_m1; Mm00664865_m1; Mm00445320_m1; Mm00493794_m1; Mm00557717_m1; Mm00508001_m1; Mm00504621_m1; Mm00607939_s1). Custom made probes to detect differentially edited 5-HT_2C_ isoforms (Table 1), were also supplied by Applied Biosystems and designed according to a recently described method [42]: 5-HT_2C_-_INI_ (non edited form), probe = [Fam]tagcaatacgtaatcctattg [MGB/NFQ]; 5-HT_2C_-_VNV_ (ABD edited form), probe = [Fam]tagcagtgcgtaatcctgttga [MGB/NFQ]; 5-HT_2C_-_VSV_ (ABCD edited form), probe = [Fam]tagcagtgcgtagtcctgttg [MGB/NFQ]; 5-HT_2C_-_VGV_ (ABECD edited form), probe = [Fam]tagcagtgcgtggtcctgttg [MGB/NFQ] and 5-HT_2C_-_VNI_ (AB edited form), probe = [Fam]tagcagtgcgtaatcctattg [MGB/NFQ]. Reaction mix was prepared using TaqMan® Universal PCR Master Mix (Applied Biosystems, Warrington, UK). Q-PCR was carried out on the ABI7300 Real Time PCR machine (Applied Biosystems, Warrington, UK). Samples were heated to 95°C for 10 minutes, and then subjected to 50 cycles of amplification by melting at 95°C and annealing at 60°C for 1 minute. Experimental samples were run in triplicate with 1 µL cDNA per reaction. No template controls were included in each run in triplicate to check for amplicon contamination. Cycle threshold (Ct) values were normalised using β-Actin and transformed using the 2−ΔCt method [43]. Fold change of relative gene expression level compared to control animals was calculated.

**Table 1:** Major mouse hypothalamic 5-HT_2C_ mRNA isoforms analysed using pyrosequencing.

5-HT_2C_ Isoform	DNA Sequence (AnB-nnn-ECn-nn-Dnn)	Nr sequences lean	% Relative occurence	Nr sequences *ob/ob*	% Relative occurence	Total nr sequences	% Relative occurence
5HT2C (VNV) ABD edited form	GnG-nnn-AAn-nnn-Gnn	3607	17.20	3429	16.37	7036	33.58
5HT2C (VNV) AD edited form	GnA-nnn-AAn-nnn-Gnn	283	1.40	253	1.21	536	2.56
5HT2C (VNI) AB edited form	GnG-nnn-AAn-nnn-Ann	1871	8.90	2175	10.38	4046	19.31
5HT2C (VNI) A edited form	GnA-nnn-AAn-nnn-Ann	857	4.10	962	4.59	1819	8.68
5HT2C (VSV) ABCD edited form	GnG-nnn-AGn-nnn-Gnn	1278	6.10	1094	5.22	2372	11.32
5HT2C (VSV) ACD edited form	GnA-nnn-AGn-nnn-Gnn	60	0.30	63	0.30	123	0.59
5HT2C (INI) UNEDITED	AnA-nnn-AAn-nnn-Ann	720	3.40	588	2.81	1308	6.24
5HT2C (VGV) ABECD edited form	GnG-nnn-GGn-nnn-Gnn	37	0.20	25	0.12	62	0.30
5HT2C (VGV) AECD edited form	GnA-nnn-GGn-nnn-Gnn	11	0.10	6	0.03	17	0.08
5HT2C (VSI) ABC edited form	GnG-nnn-AGn-nnn-Ann	850	4.10	997	4.76	1847	8.82
5HT2C (VSI) AC edited form	GnA-nnn-AGn-nnn-Ann	119	0.60	114	0.54	233	1.11
5HT2C (MNI) B edited form	AnG-nnn-AAn-nnn-Ann	45	0.20	48	0.23	93	0.44
5HT2C (IDI) E edited form	AnA-nnn-GAn-nnn-Ann	19	0.10	12	0.06	31	0.15
5HT2C (ISI) C edited form	AnA-nnn-AGn-nnn-Ann	67	0.30	61	0.29	128	0.61
5HT2C (INV) D edited form	AnA-nnn-AAn-nnn-Ann	354	1.70	203	0.97	557	2.66
5HT2C (VDV) ABEC edited form	GnG-nnn-GAn-nnn-Gnn	98	0.50	77	0.37	175	0.84
5HT2C (VDV) AED edited form	GnA-nnn-GAn-nnn-Gnn	16	0.10	9	0.04	25	0.12
5HT2C (VDI) ABE edited form	GnG-nnn-GAn-nnn-Ann	84	0.40	70	0.33	154	0.74
5HT2C (VDI) AE edited form	GnA-nnn-GAn-nnn-Ann	26	0.10	24	0.11	50	0.24
5HT2C (ISV) CD edited form	AnA-nnn-AGn-nnn-Gnn	30	0.10	52	0.25	82	0.39
5HT2C (MNV) BD edited form	AnG-nnn-AAn-nnn-Gnn	54	0.30	41	0.20	95	0.45
5HT2C (VGI) ABEC edited form	GnG-nnn-GGn-nnn-Ann	30	0.10	29	0.14	59	0.28
5HT2C (VGI) AEC edited form	GnA-nnn-GGn-nnn-Ann	31	0.10	21	0.10	52	0.25
5HT2C (IGV) ECD edited form	AnA-nnn-GGn-nnn-Gnn	1	0.00	10	0.05	11	0.05
5HT2C (MSI) BC edited form	AnG-nnn-AGn-nnn-Ann	6	0.00	9	0.04	15	0.07
5HT2C (IGI) EC edited form	AnA-nnn-GGn-nnn-Ann	6	0.00	2	0.01	8	0.04
5HT2C (IDV) ED edited form	AnA-nnn-GAn-nnn-Gnn	14	0.10	3	0.01	17	0.08
5HT2C (MDI) BE edited form	AnG-nnn-GAn-nnn-Ann	0	0.00	0	0.00	0	0.00
5HT2C (MGI) BEC edited form	AnG-nnn-GGn-nnn-Ann	0	0.00	0	0.00	0	0.00
5HT2C (MDV) BED edited form	AnG-nnn-GAn-nnn-Gnn	0	0.00	0	0.00	0	0.00
5HT2C (MSV) BCD edited form	AnG-nnn-AGn-nnn-Gnn	0	0.00	0	0.00	0	0.00
5HT2C (MGV) BECD edited form	AnG-nnn-GGn-nnn-Gnn	0	0.00	0	0.00	0	0.00

### Sequence analysis

Direct sequencing of 5-HT_2C_ receptor transcripts was performed after amplification of the editing cassette of the 5-HT_2C_ receptor. The editing cassette was amplified with PCR using the following primer sets: Editing cassette sense; 5′-TGCTGATATGCTGGTGGGACT-3′, Editing cassette antisense; 5′-TCGTCCCTCAGTCCAATCACAG-3′. PCR products were run on a 2% agarose gel to reduce background on sequencing chromatogram. Expected bands (∼300 bp) were isolated and purified using Purelink gel extraction kit (Invitrogen) according to manufacturer's instructions. Purified amplicons were eluted in 20 ul elution buffer and sent to Eurofins MWG operon for custom DNA sequencing using primer Editing sequence antisense; 5′-GATATTGCCCAAACGATGGC-3′. Sequencing chromatograms were aligned using Clustal W and raw relative peak amplitude data for each sample was analyzed. Editing frequency was quantified comparing the height of the adenosine and guanosine peaks on the sequencing chromatogram. Gross editing frequency was calculated using the following formula: X = G height/(A height + G height). The real editing frequency was calculated following the calibration quotation: A site; Y = 1.114*X, B site; 1.009*X. Pyrosequencing analysis of the 5-HT_2C_ receptor RNA editing profiles were performed using next generation 454-sequencing. Briefly, the 5-HT_2C_ editing cassette was PCR purified using similar primers as described above with the addition of an adaptor, designated adaptor A (5′-CGTATCGCCTCCCTCGCGCCATCAG-3′) in forward primer as well as barcode 1 for lean control animals (ACGAGTGCGT) and barcode 2 for *ob/ob* animals (ACGCTCGACA). In addition, a reverse primer, similar as above, was used including an adaptor, adaptor B (5′ CTATGCGCCTTGCCAGCCCGCTCAG-3′). Bands of correct size (∼300 bp) were isolated and purified using Purelink gel extraction kit (Invitrogen) according to manufacturer's instructions. Following gel purification, PCR products were precipitated with sodium acetate to remove chaotropic salts. PCR products from *ob/ob* (n = 8) and lean control (n = 8) were pooled, respectively, and PCR product was sent on dry ice to Roche (Branford CT USA) for 454-sequencing on a Roche 454 GS-FLX using Titanium chemistry.

### Statistical Analysis

Results for body weight and food intake are expressed as mean ± SEM. A two-way repeated measures ANOVA was used where appropriate with planned comparisons. Analysis of mRNA expression levels is depicted as fold change compared to control. Gene expression data are presented as the mean values ± SEM. Two-tailed unpaired Student's t-test were used to compare baseline values in obese and lean animals with a correction for multiple tests. The statistical significance was indicated as follows: * indicates p<0.05; ** indicates p<0.01 and *** indicates p<0.001. For the pyrosequencing dataset, the sequenced cDNA amplicons were quality filtered using Lucy software with the defaults for maximum acceptable average probability of error (0.025) and the maximum probability of error that is allowed for the 2 bases at each end (0.02). Sequences were aligned with MUSCLE (version 3.8.31) [44]. Identification of differentially associated RNA editing sites was carried out by assigning sequences based on the associated barcode, using the barcode identifiers. Frequencies of each base at the sites of interest were analysed using the Fisher's exact test with Bonferroni correction.

## Results

### Food intake and body weight

**Figure 2:**
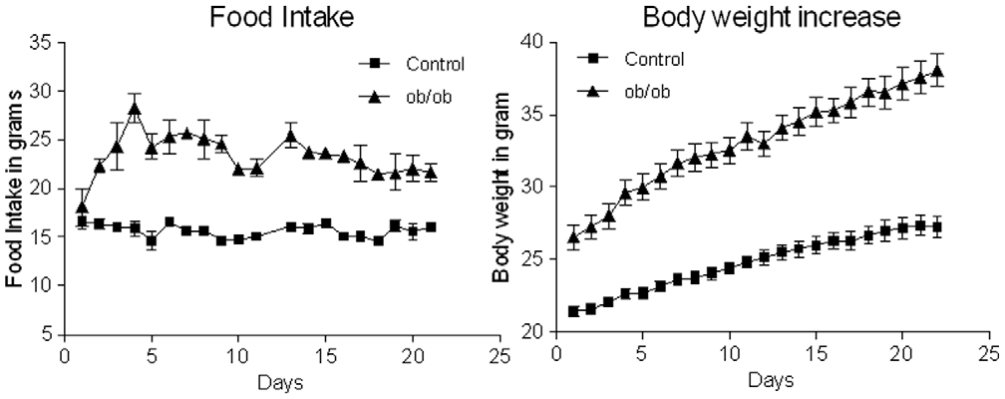
Body weight and food intake in mouse model of obesity. A) Repeated measures ANOVA showed significant increase in body weight in *ob/ob* mice; F(1;14) = 66.421;p<0.001. B) Food intake was significantly higher in *ob/ob* mice compared to lean control as analysed using repeated measures ANOVA; F(1;2) = 52.333;p<0.001; n = 8 per genotype.

In our experiments, the leptin-deficient *ob/ob* mice were hyperphagic, consuming 50% more food compared to their controls during *ad libitum* conditions, and displayed significantly higher body weights characteristic of the obesity phenotype (Figure 2A and B). When analysing body weight, repeated measures ANOVA showed significant main effect of genotype; F(1;14) = 66.421;p<0.001, as well as a significant interaction of day and genotype; F(1.938;27.135) = 33.589;p<0.001, and a significant main effect of day: F(1.938;27.135) = 420.308;p<0.001. In addition, food intake analysed using repeated measures ANOVA showed a significant main effect of genotype; F(1;2) = 52.333;p<0.001, as well as a significant interaction of day and genotype; F(17.904;35.808) = 5.993;p<0.001, and a significant main effect of day: F(17.904;35.808) = 5.081;p<0.001.

### Serotonin turnover

**Figure 3:**
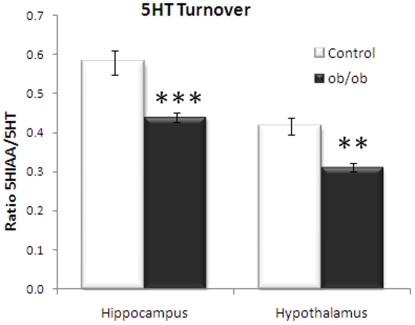
Monoamine analysis in brain regions. Decreased serotonin turnover is observed in hippocampus and hypothalamus of *ob/ob* mice compared to control. Unpaired, two-tailed T-test; statistical significance is notated as *** p<0.001, ** p<0.01 compared to lean control; n = 8 for hypothalamus, n = 10 for hippocampus.

Serotonin levels and serotonin metabolites were analysed in a different cohort of animals in the hypothalamus and hippocampus (Figure 3). No changes in 5-HT levels could be detected in *ob/ob* mice compared to control (data not shown). However, an overall decrease in the 5HIAA levels was observed (data not shown) leading to significant decrease in 5HIAA/5HT ratio in *ob/ob* mice in hippocampus (p<0.001) and hypothalamus (P<0.01) compared to lean control littermates. The hypothalamus is the main processor and integrator of peripheral metabolic information controlling food intake and plays a key role in the homeostatic regulation of appetite and energy metabolism [3], [45]. The hippocampus, a brain structure involved in learning and memory function, has recently been linked with food intake control [46].

### Serotonergic receptor mRNA expression

**Figure 4:**
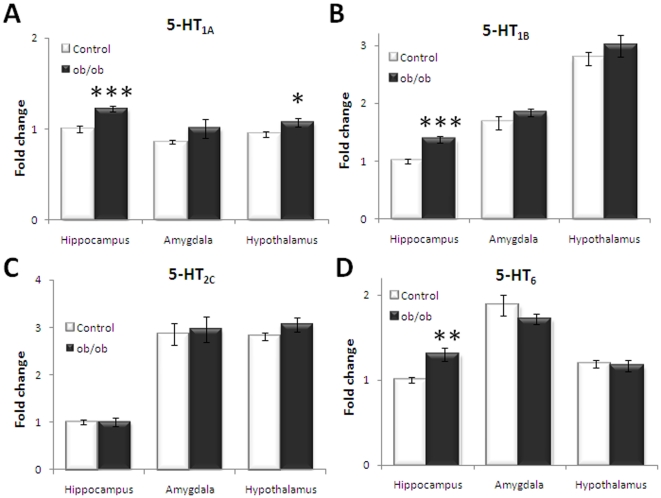
Central serotonin (5-HT) receptor mRNA expression. A) 5-HT_1A_ mRNA is increased in *ob/ob* mice in hippocampus and hypothalamus. B) 5-HT_1B_ mRNA is increased in *ob/ob* mice in hippocampus. C) 5-HT_6_ mRNA is increased in *ob/ob* mice in hippocampus. D) No change in mRNA levels of total 5-HT_2C_ mRNA measured using qRT-PCR relative to β-actin expression. Fold changes depicted compared to hippocampus in control group. Unpaired, two-tailed T-test; statistical significance is notated as *** p<0.001, ** p<0.01, * p<0.05 compared to lean control; n = 7–8 per genotype.

**Figure 5:**
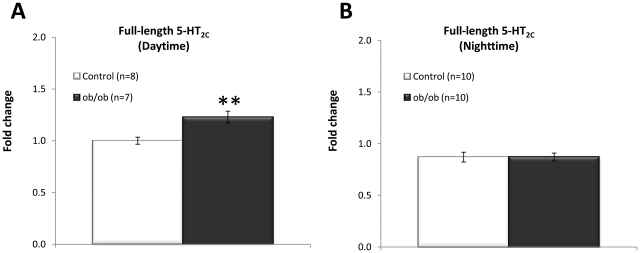
Daytime and nighttime full-length 5-HT_2C_ receptor mRNA expression in the hypothalamus. A) Significantly increased expression of the full-length 5-HT_2C_ receptor in hypothalamus of *ob/ob* mice was observed in daytime. B) No change in expression of the full-length 5-HT_2C_ receptor was observed in animals culled in nighttime. mRNA measured using qRT-PCR relative to β-actin expression. Unpaired, two-tailed T-test; statistical significance is notated as ** p<0.01, compared to lean control; n = 7–10 per genotype.

To determine central serotonergic receptor expression in relation to the obesity phenotype, hypothalamic receptor mRNA expression was analysed using quantitative real-time PCR together with mRNA levels in the hippocampus and amygdala. The hippocampal 5-HT_1A_ (p<0.001), 5-HT_1B_ (p<0.001) and 5-HT_6_ (p<0.01) were significantly increased in obese, leptin-deficient mice compared to their lean counterpart control (Figure 4A, B and C). On the hypothalamic level, only the 5-HT_1A_ receptor of 5-HT receptors analysed demonstrated a significant (p = 0.042) increased expression in obese mice compared to lean control (Figure 4A). Total 5-HT_2C_ mRNA expression was analysed using a probe spanning the exon 3 and 4 boundary of translated mRNA, detecting the full-length 5-HT_2C_ receptor expression as well as expression of all splice variants. No differential expression of total 5-HT_2C_ receptor mRNA expression was observed between *ob/ob* and control groups in all regions assessed (Figure 4D). However, when analysing 5-HT_2C_ receptor mRNA expression levels using a probe spanning the exon 5 and 6 boundary which solely detects full-length 5-HT_2C_ receptor mRNA, a significant (p = 0.004) increase in expression of full-length 5-HT_2C_ mRNA was observed in the hypothalamus of obese mice relative to the lean mice (Figure 5A). No difference in full-length 5-HT_2C_ mRNA expression was observed in the hippocampus or amygdale (data not shown).

### Hippocampal 5-HT_2C_ receptor editing

**Figure 6:**
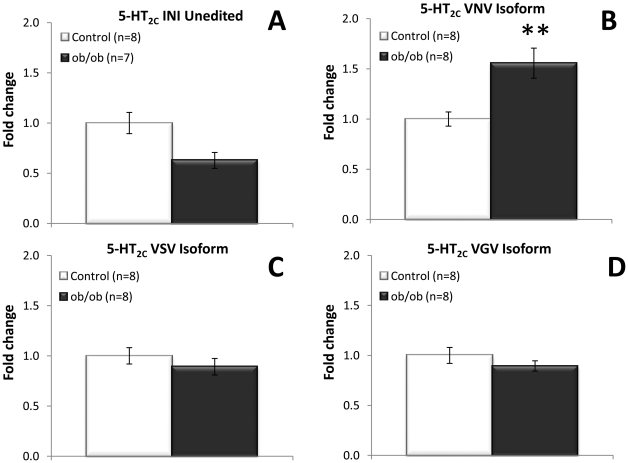
5-HT_2C_ receptor editing in the hippocampus. Expression of 5-HT_2C_ receptor isoforms in order of fully unedited isoform to fully edited 5-HT_2C_ receptor isoform. A) Decreased expression of the unedited 5-HT_2C_-INI isoform in hippocampus of *ob/ob* mice, but not statistically significant. B) Significantly increased expression of the edited 5-HT_2C_-VNV (ABD edited) isoform in hippocampus of *ob/ob* mice. C) No change in mRNA levels of 5-HT_2C_-VSV (ABCD edited) isoform. D) No change in mRNA levels of the 5-HT_2C_-VGV (ABECD edited) isoform. All mRNA measured using qRT-PCR relative to β-actin expression and depicted as fold change compared to lean control littermates. Unpaired, two-tailed T-test; statistical significance is notated as ** p<0.01, compared to lean control; n = 7–8 per genotype.

**Figure 7:**
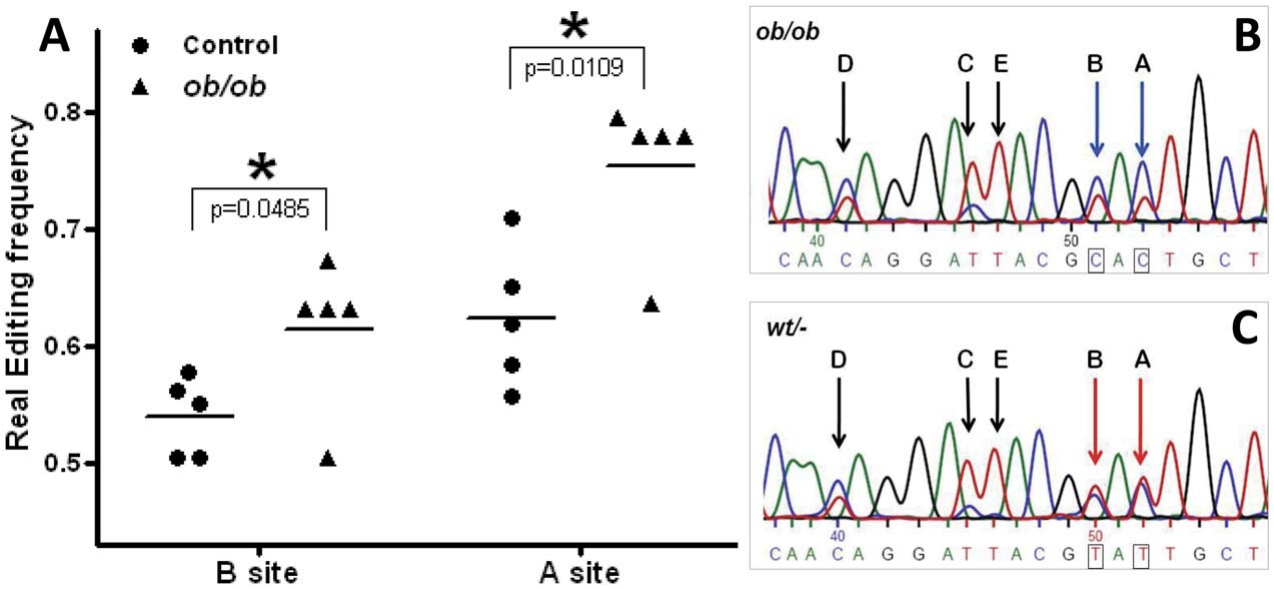
5-HT_2C_ receptor editing in the hippocampus. Editing of hippocampal the 5-HT_2C_ receptor was pinpointed to nucleotide position A and B using direct sequencing. A) Column scatter plot of editing frequencies on site A and B of the editing cassette. B) A typical reverse complement chromatogram trace of an *ob/ob* mouse is depicted. Specific editing positions A to E are indicated by arrows. C) A typical control chromatogram is depicted. Unpaired, two-tailed T-test; statistical significance is notated as * p<0.05, compared to lean control; n = 5 per genotype.

Editing of the 5-HT_2C_ receptor relative to total 5-HT_2C_ mRNA levels in the hippocampus was analysed by a recently described real-time PCR method using a 5-HT_2C_ probes specific for several edited 5-HT_2C_ isoforms, all expressed in mouse brain [26], [42]. Specific significantly increased expression of the 5-HT_2C_-VNV isoform (ABD edited), indicative of increased editing, was observed in the hippocampus (p = 0.005) of *ob/ob* mice compared to lean control (Figure 6B). A numerical decrease in mRNA levels of the unedited 5-HT_2C_-INI isoform was noted in *ob/ob* mice compared to control, but this effect was not statistically significant (Figure 6A). No difference was observed when analysing mRNA expression with the 5-HT_2C_-VSV (ABCD edited) or the 5-HT_2C_-VGV (ABECD edited) probe (Figure 6C and 6D). No differential editing of 5-HT_2C_ receptor in amygdala was found (data not shown). In addition, the 5-HT_2C_ receptor editing frequency was analysed using a direct sequencing method, pinpointing the change in 5-HT_2C_ editing to position A and B of the editing cassette (Figure 7), which corresponds to the isoform detected with the 5-HT_2C_-VNV (ABD edited) probe.

### Hypothalamic 5-HT_2C_ receptor editing

**Figure 8:**
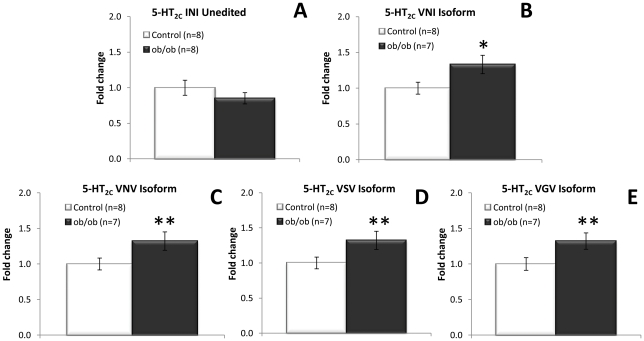
5-HT_2C_ receptor editing in the hypothalamus. Expression of 5-HT_2C_ receptor isoforms in order of fully unedited isoform to fully edited 5-HT_2C_ receptor isoform. A) No change in expression of the unedited 5-HT_2C_-INI isoform. B) Significantly increased expression of the edited 5-HT_2C_-VNI (AB edited) isoform in hypothalamus of *ob/ob* mice. C) Significantly increased expression of the edited 5-HT_2C_-VNV (ABD edited) isoform in hypothalamus of *ob/ob* mice. D) Significant increase in expression of mRNA levels of 5-HT_2C_-VSV (ABCD edited) in *ob/ob* mice compared to control. E) Significant increase in expression of mRNA levels of 5-HT_2C_-VGV (ABECD edited) isoform. All mRNA measured using qRT-PCR relative to β-actin expression and depicted as fold change compared to lean control littermates. Unpaired, two-tailed T-test; statistical significance is notated as ** p<0.01, * p<0.05 compared to lean control; n = 7–8 per genotype.

In the hypothalamus, using quantitative real-time PCR, a significant increase in 5-HT_2C_ mRNA editing in *ob/ob* mice compared to control was shown for all edited 5-HT_2C_ isoforms tested (Figure 8). However, no difference in editing could be observed for the 5-HT_2C_ receptor using direct sequencing (data not shown). This apparent contradiction between sequencing results and quantitative real-time PCR outcome may be explained by the observed increased mRNA levels of full-length 5-HT_2C_ (Figure 5A). Full-length 5-HT_2C_ can be edited and therefore an increase of all major 5-HT_2C_ isoforms, including the low abundant isoforms, may merely reflect an increase of full-length 5-HT_2C_ receptor. This concept is reinforced by the observed lack of an increase in full-length 5-HT_2C_ mRNA levels observed in the evening (Figure 5B) coupled with unchanged mRNA levels of 5-HT_2C_ isoforms (data not shown). To more precisely pinpoint if hypothalamic 5-HT_2C_ receptor editing is affected in obese versus lean mice, samples were analysed using pyrosequencing, which is a more sensitive and quantitative method of sequencing. In pyrosequencing, approximately equal amounts of cDNA amplicons from *ob/ob* mice (14763) compared to lean control (14958) were sequenced, with a combined total of 29721 reads. After sequence validation and filtering, a total of 20951 sequences, comprising both lean control and *ob/ob* 5-HT_2C_ editing cassette sequences were passed and aligned accordingly. Pyrosequencing demonstrated the 5-HT_2C_–VNV (ABD/AD edited), 5-HT_2C_–VNI (AB/A edited) and the 5-HT_2C_–VSV (ABCD/ACD edited) isoforms to be indeed the major isoforms, in decreasing order of occurrence (Table 1). The fully edited isoform, 5-HT_2C_-VGV (ABECD/AECD edited) was one of the least abundantly expressed isoforms. Specific 5-HT_2C_ RNA residues in the pooled *ob/ob* group were compared to the pooled lean control group and pinpointed an increase in editing on position A (p = 2.07*10^−8^) and a decrease in editing on position D (p = 4.47*10^−11^) in *ob/ob* mice compared to control (Table 2). This small but significant change of editing corresponds to an increase of the 5-HT_2C_-VNI isoform in *ob/ob* mice compared to lean counterpart (Table 1). The 5-HT_2C_ receptors are widely expressed in the hypothalamus, well beyond the pro-opiomelanocortin (POMC) expressing neurons of the arcuate nucleus regulating feeding behaviour, and this small increase in 5-HT_2C_–VNI isoform may well be diluted by other 5-HT_2C_ receptor expressing nuclei in the hypothalamus.

**Table 2:** Site-specific hypothalamic 5-HT_2C_ mRNA editing in *ob/ob* mice compared to control.

Edit site percentage (%)	A	B	E	C	D
Lean Control (n = 8)	87.58	75.23	3.67	24.39	55.18
*ob/ob* (n = 8)	90.01	76.67	3.01	23.7	50.65
Δ (%)	↑2.43	↑1.44	↓0.66	↓0.69	↓4.5
P-value	2.07*10^−8^	-	-	-	4.47*10^−11^

### Hypothalamic adenosine deaminase mRNA levels and effect of time

**Figure 9:**
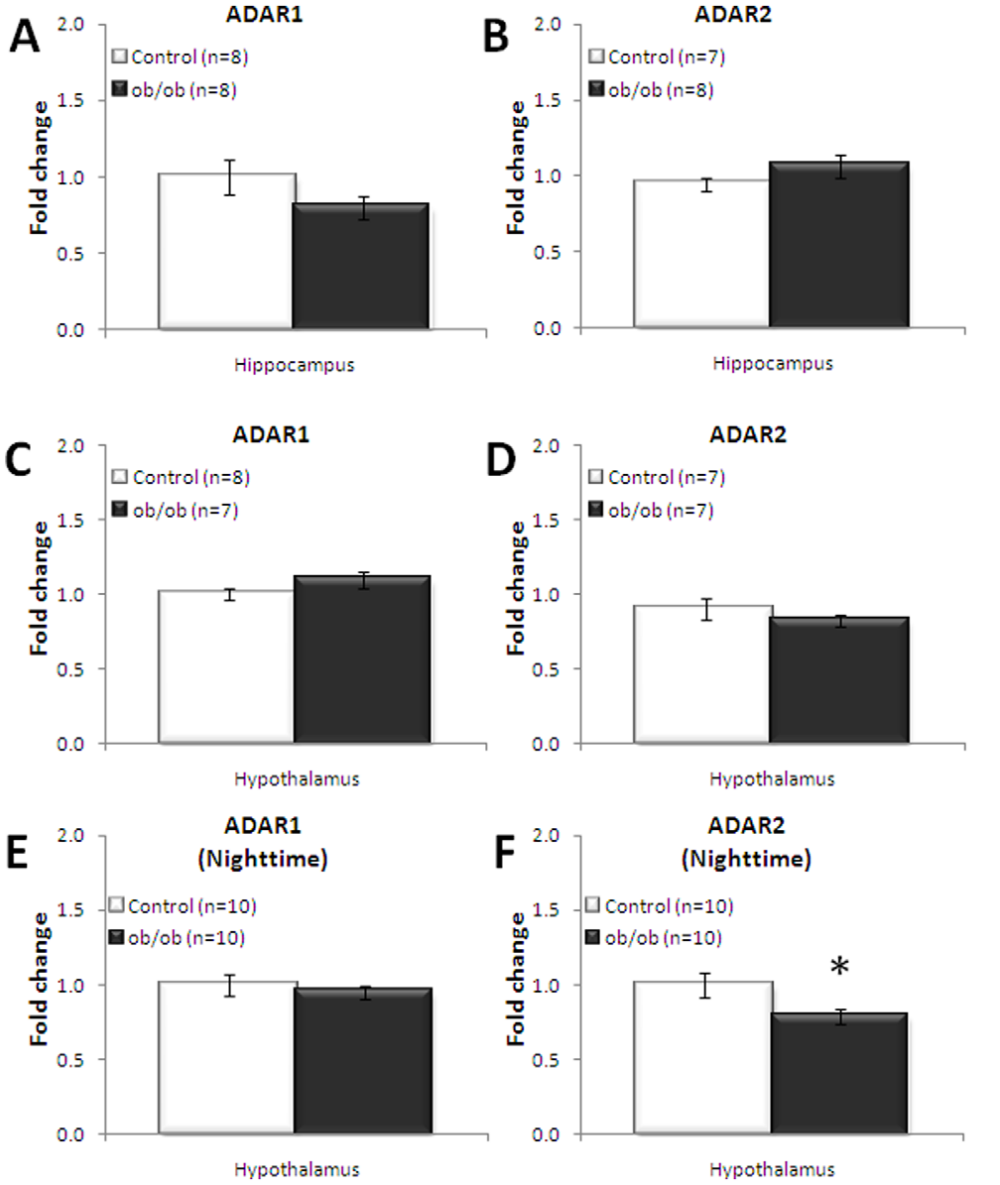
Adenosine deaminase (ADAR) mRNA expression. No significantly increased expression of the adenosine deaminase, ADAR1 (A) or ADAR2 (B) in the hippocampus. No significantly increased expression of the hypothalamic adenosine deaminase, ADAR1 (C) or ADAR2 (D) in hypothalamus during the day. No significantly increased expression of the hypothalamic adenosine deaminase, ADAR1 (E) at nighttime. However, ADAR2 mRNA levels at nighttime are significantly reduced in hypothalamus of *ob/ob* mice (F). All mRNA is measured using qRT-PCR relative to β-actin expression. Unpaired, two-tailed T-test; statistical significance is notated as * p<0.05 compared to lean control; n = 7–10 per genotype.

In samples harvested during the daytime, no significant alterations in adenosine deaminase acting on RNA (ADAR1 and ADAR2), the enzymes responsible for editing, were observed in either hippocampus or hypothalamus (Figure 9 A to D). However, a decrease of ADAR1 mRNA expression was observed in hippocampal tissue of *ob/ob* mice compared to lean control, but this did not reach statistical significance. Interestingly, a similar decrease in ADAR2 expression, which did reach significance (p<0.05), was detected in the hypothalamus of obese mice relative to lean mice in the evening, before the active phase of food intake (Figure 9F). Interestingly, a significant increase in ADAR1 (p = 0.005) was detected in the amygdala of obese mice relative to lean mice (data not shown).

## Discussion

The importance of the central serotonergic system, including the 5-HT_2C_, 5-HT_1B_ and 5-HT_6_ receptors, in the regulation of feeding behaviour, body weight and energy homeostasis has been consistently demonstrated in pharmacological and genetic studies [7], [9], [12], [47]. This study demonstrates significant increases in hypothalamic 5-HT_1A_ and 5-HT_2C_ receptor mRNA expression as well as in hippocampal 5-HT_1A_, 5-HT_1B_ and 5-HT_6_ receptor expression in obese mice (*ob/ob*) compared to lean control. Moreover, editing of the 5-HT_2C_ receptor on specific nucleotide positions was detected in the hippocampus as well as in hypothalamic tissue. In conclusion, we suggest that 5-HT_2C_ receptor mRNA expression changes and 5-HT_2C_ receptor editing may play a key role in the observed hyperphagic phenotype in the leptin-deficient obese mouse model.

### Serotonin 2C receptor mRNA expression

Hypothalamic full-length 5-HT_2C_ receptor mRNA levels were increased in obese mice relative to lean controls, which reinforce the key role of the 5-HT_2C_ receptor in the regulation of food intake and appetite. Previously, it has been shown that 5-HT_2C_ receptor mutants are hyperphagic leading to an obese phenotype and impaired glucose tolerance [48]. In addition, hyperphagia in A(y) mice with increased expression levels of the agouti peptide has been associated with increased hypothalamic 5-HT_2C_ expression [49]. The increased hypothalamic full-length 5-HT_2C_ receptor expression in obese mice was observed only in samples harvested after the active phase of the animals (daytime samples), while in samples taken at the onset of the dark phase (nighttime) no altered full-length 5-HT_2C_ receptor expression was observed. The nighttime is the active phase of the mouse where baseline food intake is greater. We therefore hypothesize that the increase in hypothalamic full-length 5-HT_2C_ in the *ob/ob* mice may occur as a compensatory mechanism during the active phase of food intake in an attempt to increase the 5-HT_2C_ mediated satiety signalling and curb the phenotypical associated hyperphagia.

### Serotonin 2C receptor editing

Considering recent data, demonstrating that that feeding behaviour and fat mass are altered in mice engineered to express a fully edited 5-HT_2C_ receptor isoform [24], [27], [28], we found it important to investigate whether 5-HT_2C_ receptor editing was affected in a physiological model of obesity. We therefore set out to determine the expression of specific edited isoforms of the 5-HT_2C_ receptor using specific probes detecting the major editing variants of the 5-HT_2C_ receptor in the hippocampus and hypothalamus. In addition, we employed direct sequencing to pinpoint the exact editing position. Noteworthy, the employment of direct sequencing to quantify RNA editing has its limitations as the height of peaks depicting the same nucleotide can differ within a chromatogram, although we found nucleotide height to be consistent based on position. Therefore, this technique requires careful interpretation and should be used in support of other methods, such as the qRTPCR employed here. This study demonstrated altered 5-HT_2C_ receptor editing in both the hippocampus and the hypothalamus of the obese mice model. Increased 5-HT_2C_ editing in the hippocampus could be pinpointed to position A and B of the 5-HT_2C_ receptor editing cassette. A recent study showed that hippocampal leptin signalling reduced food intake and that ventral hippocampal leptin signalling contributes to the inhibition of food-related memories elicited by contextual stimuli [46] indicating a key role for hippocampal mediated regulation of food intake. The absence of hippocampal leptin signalling in the leptin deficient *ob/ob* mice may suggest abnormal food-related memory processing to be involved in the hyperphagic phenotype (but see [50]). Our findings suggest that differential 5-HT_2C_ isoform expression potentially also plays a key role in the hippocampal mediated regulation of food intake and food-related memory processing. This hypothesis is reinforced by studies demonstrating the involvement of the 5-HT_2C_ receptor in memory function and consolidation [51], [52], [53]. However, the hippocampus is mainly involved in learning and memory and involvement of 5-HT_2C_ receptor editing within this domain of hippocampal function and particularly within psychological disorders, such as schizophrenia and seizure disorders such as epilepsy, remains to be investigated. Analysis of the editing profile of the 5-HT_2C_ receptor in the hypothalamus demonstrated a significant increase in editing on position A but a significantly decreased editing on position D, corresponding to an increased expression of the partially edited 5-HT_2C_–VNI isoform. It is tempting to speculate on the functional consequences of selective 5-HT_2C_ receptor mRNA editing in specific regions of the brain. Individual 5-HT_2C_ isoforms have shown to demonstrate differential constitutive activity, affinity, potency and a different ability to couple to G-proteins [19], [20], [21], [22], [23], [24], [25]. An increased 5-HT_2C_ receptor editing profile renders the 5-HT_2C_ receptor less functional. Thus, the increased expression of the VNI edited 5-HT_2C_ receptor isoform may point to a reduced cellular function. This supports the premise of decreased 5-HT_2C_ receptor function in reducing appetite-suppression in the *ob/ob* mouse model. However, we cannot exclude that different hypothalamic nuclei express different 5-HT_2C_ receptor editing isoforms.

Additionally, expression levels of the adenosine deaminase enzymes (ADAR1 and ADAR2), the enzymes responsible for RNA editing, were investigated. It has been shown that expression levels of both the enzymes ADAR1 and ADAR2 directly affect the RNA editing level of 5-HT_2C_ [25], [26], [54], [55], [56]. ADAR1 selectively edits the A and B sites of the 5-HT_2C_ receptor, whereas ADAR2 edits exclusively D site of the 5-HT_2C_ receptor. No differential ADAR expression were found in the hippocampus (Figure 8A and 8B) or hypothalamus (Figure 8C and 8D) of the obese mice in the daytime experiments. This may suggest that the increased 5-HT_2C_ editing in obese mice is not a consequence of altered ADAR expression but may potentially be due to other molecular mechanism, such as 5-HT_2C_ receptor splicing or degradation. Interestingly, a significant decrease in ADAR2 mRNA levels, in hypothalamic *ob/ob* mice relative to the lean control mice, in samples taken in the evening was observed (Figure 8F). Reduced ADAR2 expression may lead to a subsequent decrease in editing on position D of the hypothalamic 5-HT_2C_ editing cassette, as observed after pyrosequencing of hypothalamic 5-HT_2C_ receptor during the daytime. In conclusion, altered 5-HT_2C_ receptor editing in combination with changes in ADAR expression in *ob/ob* mice suggest a dynamic regulation in the appetite-suppressing activity of the 5-HT_2C_ receptor through receptor editing.

### Serotonin 1A, serotonin 1B and serotonin 6 receptor expression

We also showed significant increased hypothalamic 5-HT_1A_ mRNA expression levels and increases in 5-HT_1A_, 5-HT_1B_ and 5-HT_6_ receptor expression in the hippocampus of obese mice (*ob/ob*). Previously, exposure to 5-HT_1A_ receptor agonists have shown to increase food intake, which would be in line with the altered 5-HT1A receptor expression [57], [58], [59]. The 5-HT_1A_ and 5-HT_1B_ receptors have also been shown to regulate 5-HT release by a negative feedback mechanism as presynaptic autoreceptors by exerting direct inhibitory effects [59]. In addition, down-regulation of neuropeptide release involved in food intake has also been demonstrated via serotonin-mediated activation of post-synaptic 5-HT_1A_ receptors in both AgRP/NPY and POMC/CART containing neurons of the arcuate nucleus [60]. Therefore, the increased post-synaptic 5-HT_1A_ and 5-HT_1B_ expression in hypothalamus and hippocampus may lead to a decrease in terminal serotonin release and may consequently reduce serotonergic activation of anorectic pathways as previously suggested [14]. Indeed, decreased 5HIAA levels and a decreased 5HIAA/5HT ratio were observed in obese, leptin deficient mice compared to lean control littermates, indicating decreased 5HT turnover, which is supported in a previous a study by Rowland and colleagues [61]. However, 5-HT_1A_ has not been a major focus as a therapeutic target in obesity research and is implicated stronger in serotonergic regulation of anxiety and depression [62], [63]. A dysregulated serotonergic tone in the hippocampus in *ob/ob* mice might contribute to the anxiogenic phenotype observed in *ob/ob* mice compared to lean control mice [50] which warrants further investigation. The 5-HT_6_ receptor has also been implicated to play a role in the regulation of satiety and energy homeostasis. However, an effect on body weight is usually associated with antagonism of this receptor [64], [65], [66]. Overall, increased central 5-HT_1A_, 5-HT_1B_ and 5-HT_6_ receptor gene expression may contribute to the obesity phenotype by decreasing serotonergic tone leading to a decreased sensitivity towards satiety signals in the leptin-deficient *ob/ob* mice.

Together, these studies demonstrate aberrant mRNA expression changes in the 5-HT receptors studied in leptin deficient obese mice. Most interestingly, our findings suggests a diurnal hypothalamic 5-HT_2C_ receptor expression and increases in 5-HT_2C_ receptor editing in the *ob/ob* mouse model of obesity, which may have important physiological consequences to either the regulation of feeding behaviour through the modulation of 5-HT_2C_ receptor mediated appetite-suppressing effects or compensatory responses to the absence of leptin. The increase in 5-HT_2C_ receptor editing in the *ob/ob* mouse model would suggest the 5-HT_2C_ receptor editing to occur as a consequence of leptin-deficiency or as a compensatory mechanism to the phenotypical-associated weight gain or hyperphagia. However, significant reduced leptin levels have previously been associated with 5-HT_2C_ editing in mice genetically engineered to only express the 5-HT_2C_-VGV isoform, the fully edited variant of the 5-HT_2C_ receptor [27]. These mice were also hyperphagic but had reduced fat mass due to increased energy expenditure. This may suggest, a bidirectional relationship between leptin and 5-HT_2C_ receptor editing independent of body weight but directly correlating to hyperphagia. It would be interesting to investigate if 5-HT_2C_ receptor editing would still occur in absence of weight gain in the *ob/ob* leptin-deficient mouse model. In addition, it would be interesting to see if 5-HT_2C_ receptor editing profiles are dynamically regulated such the observed diurnal change in full-length 5-HT_2C_ receptor mRNA expression in this study and the time-of day dependent ghrelin receptor mRNA expression observed in our previous studies [67]. In addition, these results warrant further investigation into corresponding 5-HT_2C_ receptor protein expression following the phenotype-associated 5-HT_2C_ receptor editing. Concomitant changes in 5-HT_2C_ receptor protein expression and receptor functioning could potentially support the conclusion that 5-HT_2C_ receptor editing is associated with obesity.
